# Trends in Primary Treatment and Median Survival Among Women With Advanced-Stage Epithelial Ovarian Cancer in the US From 2004 to 2016

**DOI:** 10.1001/jamanetworkopen.2020.17517

**Published:** 2020-09-25

**Authors:** Anne T. Knisely, Caryn M. St. Clair, June Y. Hou, Fady Khoury Collado, Dawn L. Hershman, Jason D. Wright, Alexander Melamed

**Affiliations:** 1 Division of Gynecologic Oncology, Department of Obstetrics and Gynecology, Columbia University Vagelos College of Physicians and Surgeons, NewYork-Presbyterian/Columbia University Irving Medical Center, New York; 2 Division of Hematology/Oncology, Department of Medicine, Columbia University Vagelos College of Physicians and Surgeons, NewYork-Presbyterian/Columbia University Irving Medical Center, New York

## Abstract

This cohort study compares time trends in the use of neoadjuvant chemotherapy and survival rates among women with advanced-stage epithelial ovarian cancer.

## Introduction

Three published clinical trials^1,2,3^ that randomized women with advanced-stage ovarian cancer to primary cytoreductive surgery before adjuvant chemotherapy or neoadjuvant chemotherapy before interval cytoreductive surgery found no evidence of a progression-free or overall survival advantage for either approach. However, those studies did demonstrate a reduction in surgical morbidity in women assigned to neoadjuvant chemotherapy. Nonetheless, most gynecologic oncologists believe that primary surgical treatment is the preferred approach for advanced ovarian cancer.^4^ Since the publication of the first of those 3 randomized trials in 2010,^1^ the use of neoadjuvant chemotherapy for ovarian cancer has increased in the US.^5^ The objective of this study was to examine and compare time trends in the use of neoadjuvant chemotherapy and the median survival among women with advanced-stage epithelial ovarian cancer in the United States.

## Methods

The Columbia University institutional review board deemed this cohort study exempt from informed consent and review because it used an existing data set without patient identifiers. We used the National Cancer Database, a cancer registry maintained by the American College of Surgeons’ Commission on Cancer and the American Cancer Society, to identify women who received treatment (ie, chemotherapy, surgery, or both) for stage IIIC or IV epithelial ovarian cancer that was diagnosed from January 1, 2004, through December 31, 2016. We calculated the proportion of women diagnosed each year (with 95% CIs) who were treated with chemotherapy as the first treatment modality (neoadjuvant chemotherapy). The primary survival outcome was time from diagnosis to death or last follow-up. For women with mature survival data (ie, those who were diagnosed 2004 to 2013), we calculated median overall survival with 95% CIs using the Kaplan-Meier method for women diagnosed in each year. Using the Bayes information criteria to identify the number and location of join points, we fitted weighted log-linear join point models to evaluate trends in use of neoadjuvant chemotherapy and median survival. The analysis was performed using the most recent National Cancer Database data (ie, diagnoses 2004 to 2016, follow-up survival through 2017). Statistical analysis was performed in August 2019 using Stata/MP statistical software version 16 (StataCorp). Two-sided *P* < .05 was considered significant, as evaluated using Wald tests.

## Results

We identified 72 171 women treated for advanced epithelial ovarian cancer between 2004 and 2016, with mean (SD) age 63.0 (12.4) years (Table). Over the study period, 53 021 women were treated with primary cytoreductive surgery (73.5%) and 19 150 women were treated with neoadjuvant chemotherapy (26.5%). From 2004 to 2006, 2976 of 16 875 women received neoadjuvant chemotherapy (17.6%), and that proportion did not change over that period (annual percentage change, 0.5%; 95% CI, −1.3% to 2.4%; *P* = .54) (Figure, A). The frequency of primary chemotherapy increased starting in 2006, increasing by 7.9% (95% CI, 7.0% to 8.7%) per year from 2006 to 2011 (*P* for change of trend < .001). Use of neoadjuvant chemotherapy accelerated in 2011, increasing by 10.3% (95% CI, 9.1% to 11.5%) per year from 2011 to 2016 (*P* for change of trend = .01) (Figure, A). By 2016, 2481 of 5501 patients received chemotherapy as their initial treatment (45.1%).

**Table.** Patient Characteristics

Characteristic	No. (%)	
	Primary cytoreductive surgery (n = 53 021)	Neoadjuvant chemotherapy (n = 19 150)
Age, mean (SD), y	62.3 (12.6)	65.1 (11.7)
Cancer stage		
IIIC	37 176 (81.5)	8440 (18.5)
IV	15 845 (59.7)	10 710 (40.33)
Tumor type		
Serous adenocarcinoma	39 438 (75.1)	13 088 (24.9)
Endometrioid adenocarcinoma	2348 (89.4)	279 (10.6)
Clear cell adenocarcinoma	1843 (82.8)	383 (17.2)
Mucinous adenocarcinoma	1372 (82.9)	283 (17.1)
Other adenocarcinoma	8020 (61.0)	5117 (39.0)

**Figure.**
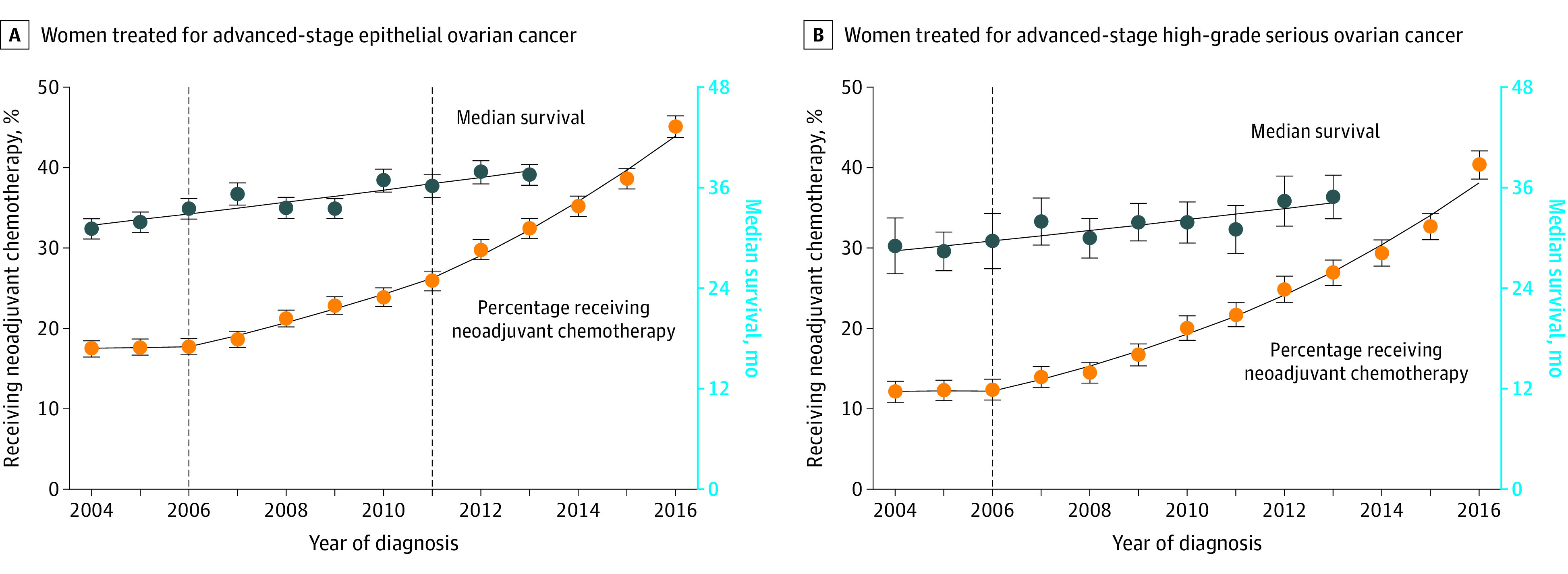
Time Trends in Use of Primary Chemotherapy and Median Survival in Commission on Cancer–accredited Cancer Centers in the United States Whiskers indicate 95% CIs; dashed vertical lines, year when statistically significant changes of trend in the use of neoadjuvant chemotherapy were identified by join point models; and solid lines, predictions from join point models.

Median survival increased from 31.1 (95% CI, 29.9 to 32.3) months in 2004 to 37.8 (95% CI, 36.4 to 38.8) months in 2013. Changing trends in the use of neoadjuvant chemotherapy during this period were not associated with a change in median survival trend, which increased by 2.1% (95% CI, 1.3% to 2.8%) per year (*P* for change of trend = .37). Results of a subgroup analysis restricted to patients with high-grade serous carcinoma were similar; increasing use of neoadjuvant chemotherapy starting in 2006 was not associated with any change of trend in median survival, which increased by 2.0% per year (95% CI, 1.2% to 2.9%) over the study period (*P* for change of trend = .21) (Figure, B).

## Discussion

This cohort study found that use of neoadjuvant chemotherapy for patients with stage IIIC and IV epithelial ovarian cancer increased from 17.6% in 2004 to 45.1% in 2016. As suggested by the results of randomized clinical trials,^1,2,3^ uptake of neoadjuvant chemotherapy was not associated with any changes in median overall survival trends, which increased over the study period. Limitations of this study include the inability to establish a causal relationship between trends in treatment and outcome and the use of a data source that is not population based.
